# Effect of Cataract Surgery on Frequency of Falls among Older Persons: A Systematic Review and Meta-Analysis

**DOI:** 10.1155/2021/2169571

**Published:** 2021-03-15

**Authors:** Luis Miguel Gutiérrez-Robledo, Miguel Angel Villasís-Keever, Arturo Avila-Avila, Raúl Hernán Medina-Campos, Roberto Carlos Castrejón-Pérez, Carmen García-Peña

**Affiliations:** ^1^Instituto Nacional de Geriatría, Mexico City, Mexico; ^2^Head of Analysis and Synthesis of Evidence Research Unit, Instituto Mexicano del Seguro Social, Mexico City, Mexico; ^3^Director of Education, Instituto Nacional de Geriatría, Mexico City, Mexico; ^4^Head of Epidemiologic and Geriatric Research, Instituto Nacional de Geriatría, Mexico City, Mexico; ^5^Health Researcher, Instituto Nacional de Geriatría, Mexico City, Mexico; ^6^Director of Research, Instituto Nacional de Geriatría, Mexico City, Mexico

## Abstract

**Background:**

Falls are a significant public health problem among older people worldwide. The aim was to perform a new systematic review and meta-analysis to assess whether cataract surgery is effective in reducing the rate of falls in older persons.

**Methods:**

The systematic review was performed following the recommendations by the Cochrane Collaboration. Original papers were included with RCT or quasi-experimental design, which described the effect on uni- or bilateral cataract surgery on the rate of falls among people aged 60 or older. Titles and abstracts were reviewed, full-text versions were retrieved, and two independent examiners reviewed them to assess inclusion criteria. All relevant variables were synthesised in an evidence table. Random-effects meta-analyses were performed pooling the trials, and results were expressed as relative risk (RR) and 95% confidence intervals.

**Results:**

The initial search reported 99 potential abstracts, and 41 full-text versions were examined. In the end, eight studies were included. Five included patients 65 years of age and older, two patients 55 years and older, and one included patients 50 years or older. Phacoemulsification and intraocular lens implant were performed in all studies. Two were RCT, and six were quasi-experimental. Falls was the main outcome. The six quasi-experimental studies reported that a reduction in the frequency of falls was observed (RR 0.68, 95% CI 0.48–0.96), although heterogeneity was significant (*I*^2^ = 74%). Only one RCT reported risk reduction of 34% (RR 0.66, 95% CI 0.45–0.96).

**Conclusions:**

This meta-analysis provides evidence that the first cataract surgery reduces the frequency of falls in older people with bilateral cataracts, but a second surgery does not have significant impact.

## 1. Background

Falls are a significant public health problem among older people worldwide. They represent the second cause of death by nonintentional injuries, with 646,000 deaths by falls occurring annually. Most of these deaths occur among persons 60 years and older. Moreover, 37.3 million nonfatal falls occur every year, producing 17 million disability-adjusted life years, mostly among older persons. [1, 2].

Factors that increase the incidence of injuries derived from falls include older age, visual impairment, cataracts (uni- or bilateral), history of falls in the two previous years, disorders of gait and balance, neurological disorders, limited mobility, back pain, limited neck rotation, cognitive impairment, dementia, and any compromise in overall capacity [3–5].

It has been suggested that visual impairment compromises the ability to avoid obstacles, being cataracts and refractive error the most frequent cause of visual impairment among older people [6–8]. Age-associated cataracts remain the leading cause of visual impairment and blindness in low-and middle-income countries, including Latin America [8, 9].

Treatment for cataracts consists of the surgical extraction of the opacified crystalline and its replacement with an intraocular lens. With different surgical techniques described and different types of intraocular lenses available, cataract surgery over all is effective in improving vision and is quick, cost-effective, and safe [10].

Visual impairment has been recognised as a risk factor for falls among older people since the 1950s [11]. Nevertheless, it is not clear whether cataract surgery decreases the incidence of falls. In 2003, a prospective study by Brannan et al. showed that cataract surgery efficaciously reduced the risk of falls in older people with visual impairment [12]. Several studies have followed after that which have evaluated the impact of cataract surgery on falls in older people, including some randomised controlled trials (RCT) and even two meta-analyses. The most recent meta-analysis by Desapriya et al. included only two clinical trials and they did not find evidence that cataract surgery reduced incident falling (OR 0.81, 95% CI 0.55–1.17) [13]. The lack of association can be explained because these two clinical trials reported a different intervention; one corresponds to the first surgery and the other to the second cataract surgery. More clinical trials evaluating the impact of cataract surgery on falls have been published since 2010.

Considering the growing accessibility to cataract surgery and the critical consequences of falls in older people and the possibility that new clinical trials may modify the results previously reported, we decided to perform a new systematic review and meta-analysis to assess whether cataract surgery is effective in reducing the rate of falls in older people.

## 2. Methods

This systematic review was performed following the recommendations by the Cochrane Collaboration. Relevant studies were retrieved from Medline, EMBASE, and Cochrane Library, using the following search terms: *cataract, cataract extraction, falls, accidental falls, aged, elderly, clinical controlled trial,* and *clinical trial*. The date of the last search was March 2019.

The following inclusion criteria were used: original papers with RCT (participants were randomly allocated to intervention or comparator) or quasi-experimental design (randomization process was not used for the allocation of the intervention, such as before-and-after studies: participants were their own controls; that is, the incidence of falls were compared before and after the intervention) which described the effect on uni- or bilateral cataract surgery on the rate of falls among people aged 60 or older. The language was restricted to English and Spanish.

### 2.1. Study Selection Process

Titles and abstracts were assessed by two independent examiners, who determined whether studies met inclusion criteria. Studies were included only when the two examiners agreed. In case of discordance, a consensus decision was made to include or exclude the studies.

Full-text versions were retrieved for all selected studies. Two independent examiners reviewed them to assess the inclusion criteria. Studies were included when both examiners agreed, and a consensus was made in case of disagreement. After this selection, relevant data were extracted from the studies, and each independent examiner performed an individual assessment of the quality of the studies.

### 2.2. Statistical Analysis

For each study included in the systematic review, all relevant variables were collected and synthesised in an evidence table. Random-effect meta-analyses were performed pooling the trials that assessed participants in a similar fashion, and results were expressed as relative risk (RR) and 95% confidence intervals. All analyses were performed using the RevMan 5.3 software.

## 3. Results

### 3.1. Study Selection Process

**Figure 1:**
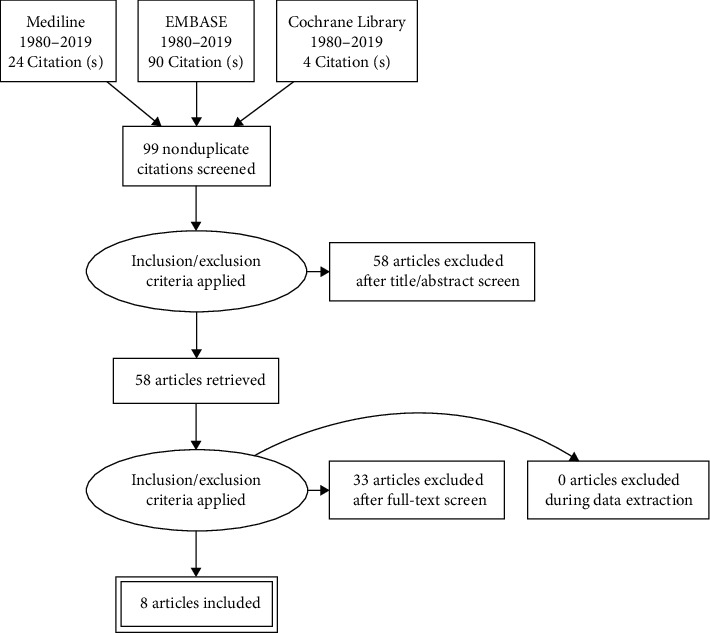
Study selection process.

Figure 1 illustrates the process of study selection. The initial search rendered 99 potential abstracts, of which 58 were excluded for not fulfilling inclusion criteria. Further on, 41 full-text versions were examined and 33 of them excluded because the design was not RCT or quasi-experimental or because they did not include falls as an outcome. In the end, eight studies were included in the systematic review [12, 14–20].

### 3.2. Study Characteristics

The eight included studies were published between 2003 and 2018. Four of them were performed in the United Kingdom [12, 15, 16, 19], two in Australia [14, 18], one in the United States [17], and one in Vietnam [20].

#### 3.2.1. Patient Characteristics

Five studies included patients 65 years of age or older [12, 15, 16, 18, 19], two included patients 55 years or older [14, 17], and one included patients 50 years or older [20]. Two studies were performed exclusively in women [15, 16], while the rest included patients of both sexes.

#### 3.2.2. Type of Surgery

In all studies, cataract surgery was performed by phacoemulsification and intraocular lens implant. The study [15] by Foss et al. included patients undergoing surgery in the second eye. Two studies included participants undergoing two surgeries (first and second eyes, with no specified time between surgeries) [14, 20]. All others included patients undergoing their first cataract surgery.

#### 3.2.3. Design

Only two studies were RCT's comparing expedite cataract surgery with the usual waiting time [15, 16]. The other six studies were quasi-experimental, comparing the incidence of falls before and after surgery.

#### 3.2.4. Falls Assessment

All studies provided a definition of the occurrence of falls. In general, a fall was defined as an unexpected or accidental event in which the participant had reached a ground level from an upper position. Four studies used the same definition as that by Lamb et al. in 2005 [21].

The specific method for assessing falls varied across the studies. Four studies used daily logs and direct interviews at the end of follow-up [12, 14, 18, 19]. The other four studies used direct interviews at the end of the follow-up period, which ranged from 6 to 12 months [15–17, 20].

### 3.3. Quality Assessment of the Studies

For the six quasi-experimental studies, the quality was assessed using the ROBINS-1 tool for risk of bias assessment (Supplementary Table 1). No risk of bias was found regarding the intervention. As for participant selection, two studies had no risk of bias [14, 18], but the rest did not provide sufficient detail of the selection procedure. None of the studies considered the possibility that falls could have been due to factors other than visual impairment. In the studies where two cataract surgeries were performed, it was not specified whether a fall assessment was performed in between surgeries [14, 20]. Regarding the two RCT's included in this systematic review [15, 16], no risk of bias was detected after applying the Cochrane risk of bias tool (Supplementary Table 2).

### 3.4. Falls after Surgery vs. No Surgery

The two RCT's included in this systematic review had a similar design comparing the rate of falls after cataract surgery in a group that underwent expedite surgery versus the rate of falls in a control group with usual waiting time to surgery (Supplementary Table 3). However, one of the studies included patients undergoing their first cataract surgery [16], and the other one included patients undergoing surgery in their second eye [15].

The study by Harwoodet al. [16] compared 154 patients undergoing cataract surgery to 152 patients with surgery delayed up to 13 months. After 12 months, 49.3% of patients in the surgery group had fallen at least once, compared to 45.3% in the control group. However, patients having two or more falls were less in the surgery group (28 frequent fallers, 18%) than in the control group (38 frequent fallers, 25%). According to the authors, the total risk reduction was 34% (RR 0.66, 95% CI 0.45–0.96).

In the study by Foss et al. [15], they compared 120 patients who had cataract surgery with 119 who had no surgery. No statistical difference was found between the two groups after 12 months (RR 0.68, 95% CI 0.39–1.19).

McGwin et al. [17] studied 122 patients who had cataract surgery on one or two eyes and compared them to 92 patients who chose not to have surgery. After 12 months, no difference was found between both groups (RR 0.96, 95% CI 0.64–1.42).

### 3.5. Frequency of Falls before Vs. after the First Cataract Surgery

**Figure 2:**
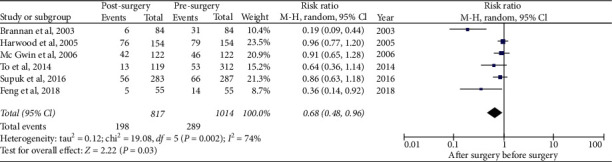
Meta-analysis of the frequency of falls after the first cataract surgery in older people.

Table 1 presents the synthesis of six quasi-experimental studies comparing [12, 14, 16, 17, 19, 20] the frequency of falls before and after cataract surgery. A meta-analysis was performed pooling 1014 patients before surgery and 817 patients after surgery. A reduction in the frequency of falls was observed after surgery (RR 0.68, 95% CI 0.48–0.96), although heterogeneity was significant (*I*^2^ = 74%) (Figure2).

Because the authors of the study (Palagy et al.) [18] did not report specific data on the incidence of falls, we could not integrate the results in the meta-analysis. However, the results have a similar trend. This study recruited 329 patients and followed a total of 228 for 12 months after cataract surgery. Before surgery, the observed rate of falls was 1.17 falls per year-person (95% CI 0.93–1.46). After surgery, the rate dropped to 0.88 falls per year-person (95% CI 0.66–1.17). They reported an annual reduction of 33% in the rate of falls after adjusting for age, sex, physical activity, and medication use (*p*=0.001).

### 3.6. Frequency of Falls before vs. after the Second Cataract Surgery

Three studies assessed falls after cataract surgery in the second eye [14, 15, 20]. It should be noted that in two of these studies, analysis of the incidence of falls was performed after the first and second surgeries. However, Feng et al. [14] managed to follow-up all 55 patients in both periods, while To et al. [20] only evaluated 193, out of a total of 413, who had initially been recruited.

**Figure 3:**
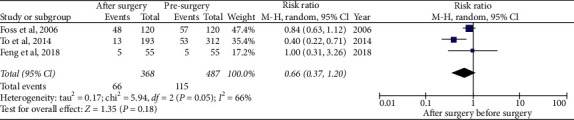
Meta-analysis of the frequency of falls after the second cataract surgery in older people.

The meta-analysis included these three studies, pooling 368 patients before surgery and 487 patients after surgery (Table 2). No statistically significant difference was observed in the risk for falling (RR 0.66, 95% CI 0.37–1.20), but heterogeneity was detected (*I*^2^ = 66%) (Figure 3).

## 4. Discussion

With the results of this systematic review and meta-analysis aimed to assess whether cataract surgery is effective in reducing the rate of falls in older people, for the first time, it can be established that cataract surgery reduces the incidence of falls in older patients with bilateral cataracts, by approximately a third. However, when considering the second cataract surgery, no significant difference in fall rates was found.

Falls in older people are usually multifactorial in origin. It is not surprising that interventions that have proven to be effective in reducing falls in older people are also multicomponent (i.e., comprised of several combined interventions aimed at multiple risk factors). Vision, however, is usually regarded as one of the most critical factors regarding falls. Age-associated visual impairment in stereopsis and contrast perception may compromise older persons' ability to avoid obstacles successfully. As such, it is reasonable to assume that the correction of visual impairment derived from cataracts might reduce the risk of falling.

We found that the first cataract surgery reduces the frequency of falls by 34%, while surgery in the second eye had no significant impact on falls according to our findings, which could explain why Foss et al. [15] did not find differences in the risk of falling after surgery since they included women following one successful cataract surgery. The available evidence does not support that the correction of cataracts in the second eye adds any benefit reducing the risk of falls, although one could argue that stereopsis does require input from both eyes.

It is also noticeable that both Harwood's and Foss's trials showed a significant reduction in the rate of falls per 1000 patient-days in the expedite surgery group compared to the control group. This is relevant because, in both studies, the control group were patients who also underwent surgery but waited longer for it. Waiting times varied widely in the control group, from 133 to 485 days. Given this variation, merely comparing the frequency of falls between groups might not be the best way to measure the effect of expediting surgery because the incidence of falls is time-dependent in nature. The rate of falls per 1000 patient-days might be a more appropriate measurement of the effect.

The study by McGwin et al. [17] showed no difference between surgery and no surgery in the frequency of falls. However, the results of this study must be taken with caution because the two groups might have had significant baseline differences that were not accounted by the authors. Although it is stated that all patients in the study could walk independently and had no dementia, no further description of their health status is provided. Patients who declined surgery may have done so for a number of reasons, including a worse self-perception of health status or worse socioeconomic conditions.

The possible beneficial effect of cataract surgery on reducing falls would have to be (in part) secondary to the improvement in visual acuity. Although it was not the present study's aim, we considered the change in visual acuity after surgery. Supplementary Table 4 shows that contrasting visual acuity among the included studies is challenging since each study used a different measurement methodology, and only a subsample of each study was evaluated. However, overall, there was a significant clinical improvement.

Although quasi-experimental studies provide weaker evidence than randomised controlled trials, our meta-analysis still makes a strong case for a reduction of the frequency of falls after the first cataract surgery. Additionally, our results make evident that cataract surgery is becoming increasingly accessible since the studies included in our analysis were performed in both developed [12, 14–19] and developing [20] countries, highlighting the relevance of cataract surgery from a public health perspective, which can become an effective measure to avoid falls and their complications in appropriately selected patients. A study by Tseng et al. [22] in Medicare beneficiaries aged 65 years and older with a diagnosis of cataracts showed that cataract surgery was associated with lower odds of hip fracture within one year after surgery compared to patients who did not have surgery. We did not assess hip fracture as an outcome in our study because, although it is one of the most severe consequences of falls in older people, it is not the only one.

We did not assess the cost-effectiveness of cataract surgery; however, it is crucial to include the costs derived from falling when evaluating cataract surgery's cost-effectiveness among older people. Still, our findings suggest that there may be potentially significant savings in costs derived from falling that might be attained by cataract surgery. An in-depth analysis of this matter is warranted.

Although this meta-analysis provides evidence that the first cataract surgery reduces the frequency of falls in older people, we recognise that it has limitations. The overall quality of the studies was deficient due to methodological issues. One issue is how to evaluate falls, where there may be recall bias since, in most studies, what happened the year before is questioned; while after surgery, the evaluation is prospective. The second is that among the studies, the number of patients is highly variable, as well as their follow-up. Finally, there are very few studies on the impact of second cataract surgery.

Further studies are needed that assess not only the efficacy of cataract surgery but also its cost-effectiveness as a potential intervention for reducing the risk of falls and their complications, such as hip fracture, in older people.

## Figures and Tables

**Table 1:** Characteristics of included studies of the relationship between the frequencies of falls after the first cataract surgery in older people.

Author	Design	Patients	Age	Sex	Follow-up time	Falls before surgery	Falls after surgery
Brannan et al., [12]	Before and after surgery	*N* = 84	79.8 ± 5.8	Female 54 (64.2%) Male 30 (35.7%)	6 months	31 (36.9%)	6 (7.1%)
Harwood et al., [16]	Controlled clinical trial: expedited surgery group	*N* = 154 (before surgery) *N* = 154 (after surgery)	Median 78.8Range: 79–95	Female 154 (100%)	12 months	79 (51.3%)	76 (49.3%)
McGwin et al., [17]	Before and after surgery	*N* = 122	70.9 ± 6.8	Female 71 (58.2%) Male 51 (41.8%)	12 months	46 (37.8%)	42 (34.4%)
To et al., [20]	Before and after surgery	*N* = 413 (before surgery) *N* = 119 (after surgery)	66.6 ± 7.9	Female 268 (64.9%) Male 145 (35.1%)	12 months	53 (12.8%)	13 (10.9%)
Supuk et al., [19]	Before and after surgery	*N* = 287 (before surgery) *N* = 283 (after surgery)	76.5 ± 6.3	Female 158 (55.1%) Male 129 (44.9%)	6 months	66 (22.9%)	56 (19.8%)
Palagyi et al., [18]	Before and after surgery	*N* = 228(before surgery) *N* = 196(after surgery)	75.7 ± 5.3	Female 182 (55.3%) Male 147 (44.7%)	12 months	1.17 (IC95% 0.93–1.46) year-person falls	0.88 (IC 95% 0.66–1.17) year-person falls
Feng et al., [14]	Before and after surgery	*N* = 55	73.3 ± 7.7	Female 30 (54.6%) Male 25 (45.4%)	99.6 ± 73.7 days	14 (25.5%)	5 (9.1%)

**Table 2:** Characteristics of included studies of the relationship the frequency of falls after the second cataract surgery in the older people.

Author	Design	Patients	Age	Sex	Follow-up time	Falls before surgery	Falls after surgery
Foss et al., [15]	Controlled clinical trial: expedited surgery	*N* = 120	Range:70–90 y	Female 120 (100%)	12 months	57 (47.5%)	48 (40.0%)
To et al., [20]	Before and after surgery	*N* = 413 (pre-surgery) *N* = 193 (post-surgery)	66.6 ± 7.9	Female 268 (64.9%) Male 145 (35.1%)	12 months	53 (12.8%)	13 (6.7%)
Feng et al., [14]	Before and after surgery	*N* = 55	73.3 ± 7.7	Female 30 (54.6%) Male 25 (45.4%)	283 ± 52.2 days	14 (25.5%)	5 (9.1%)

## Data Availability

The data used to support this study are available from the corresponding author upon reasonable request.

## Supplementary Materials

Supplementary Materials Supplementary Table 1: quality analysis of quasi-experimental studies: risk of bias^*∗*^. Supplementary Table 2: quality analysis of controlled clinical trials: risk of bias^∗∗^. Supplementary Table 3: studies comparing the frequency of falls between those who perform and do not perform cataract surgery in older people. Supplementary Table 4: visual acuity evaluation before and after cataract surgery.
