# Supplementary material for: Effect of Cataract Surgery on Frequency of Falls among Older Persons: A Systematic Review and Meta-Analysis
Source: J Ophthalmol. 2021 Mar 15;2021:2169571. doi: 10.1155/2021/2169571 (PMC7987466; doi:10.1155/2021/2169571)
Supplement: Supplementary Materials — Supplementary Table 1: quality analysis of quasi-experimental studies: risk of bias∗. Supplementary Table 2: quality analysis of controlled clinical trials: risk of bias∗∗. Supplementary Table 3: studies comparing the frequency of falls between those who perform and do not perform cataract surgery in older people. Supplementary Table 4: visual acuity evaluation before and after cataract surgery. [file 2169571.f1.docx]

**Supplementary Table 1.** Quality analysis of quasi-experimental studies: risk of bias*

| **Bias** | **Brannan, 2003** | **McGwin, 2006** | **To,**  **2014** | **Supuk,**  **2016** | **Palagi,**  **2017** | **Feng, 2018** |
| --- | --- | --- | --- | --- | --- | --- |
| **Pre and trans-intervention** | | | | | | |
| *Confusion* | Serious | Serious | Low | No information | Low | Low |
| *Selection* | Serious | Serious | Low | Serious | Low | Moderate |
| *Intervention* | Low | Low | Low | Low | Low | Low |
| **Post- intervention** | | | | | | |
| *Intervention deviation* | Low | Serious | Moderate | Critic | Serious | Low |
| *Lost data* | Low | Serious | Serious | Low | Low | Low |
| *Dependent variable evaluation* | Low | Moderate | Moderate | Moderate | Low | Low |
| *Incomplete report results* | Low | Low | Low | Low | Low | Low |

***Cochrane** **ROBINS-1 tool**

**Supplementary Table 2.** Quality analysis of controlled clinical trials: risk of bias **

| **Bias** | **Harwood, 2005** | **Foss, 2006** |
| --- | --- | --- |
| *Randomization process* | Low | Low |
| *Intervention deviation* | Low | Low |
| *Lost data/missing data* | Low | Low |
| *Dependent variable evaluation* | Low | Low |
| *Incomplete report results* | Low | Low |

**** Cochrane Risk of Bias Assessment**

**Supplementary Table 3.** Studies comparing the frequency of falls between those who perform and do not perform cataract surgery in elderly.

| **Author** | **Design** | **Surgery** | **Without surgery** | **Falls in the surgery group** | **Falls in control group (without surgery)** |
| --- | --- | --- | --- | --- | --- |
| **Harwood, 2005** | Controlled clinical trial:  1) **First** eye surgery  2) Without surgery (waiting list) | N = 154 | N = 152 | .376 (49%) | 69 (45.3%) |
| **McGwin, 2006** | Before and after surgery | N = 122 | N = 92 | 42 (34.4%) | 29 (31.5%) |
| **Foss, 2006** | Controlled clinical trial:  1) **Second** eye surgery  2) Without surgery (waiting list) | N = 120 | N = 119 | 48 (40.0%) | 41 (34.4%) |

**Supplementary Table 4.** Visual acuity evaluation pre and after cataract surgery.

| **Author** | **Patients evaluated** | **Method of visual acuity (VA) evaluation** | **VA before surgery**  (mean ± SD) | **VA after surgery**  (mean ± SD) |
| --- | --- | --- | --- | --- |
| **Brannan, 2003** | N = 84 | Snellen card | Unclear: authors reported 76 “*postoperative non-fallers*” patients:  ≥ 6/18: n=61  6/24-3/60: n=15 | Unclear: "88% obtained a postoperative corrected visual acuity of 6/6-6/9" |
| **Harwood, 2005** | N = 148 | Early Treatment Diabetic Retinopathy Study: logarithm of minimum angle resolvable (logMAR) units | Unaided VA: 0.51*  Spectacles VA: 0.30*  Pinhole VA: 0.21* | Unaided VA: 0.22*  Spectacles VA: 0.10*  Pinhole VA: 0.09* |
| **McGwin, 2006** | N = 122 | Early Treatment Diabetic Retinopathy Study | Better eye: 0.28 ± 0.20  Worse eye: 0.55 ± 0.24 | Better eye: 0.09 ± 0.15  Worse eye: 0.28 ± 0.21 |
| **Foss, 2006** | N = 116 | Early Treatment Diabetic Retinopathy Study (logMAR units) | Unaided VA: 0.22*  Spectacles VA: 0.09*  Pinhole VA: 0.10* | Unaided VA: 0.15*  Spectacles VA: 0.04*  Pinhole VA: 0.04* |
| **To, 2014** | N = 413 (pre-surgery)  N = 247 (post-surgery) | Snellen card, expressed on logMAR units | Binocular VA: 0.62 ± 0.41 | Binocular VA: 0.09 ± 0.19 |
| **Supuk, 2016** | N = 262 | Snellen card converted to logMAR | Operated eye:  0.30 (0.20-0.40)** | Operated eye:  0.10 (0.00-0.24)** |
| **Palagyi, 2017** | N = 196 | Early Treatment Diabetic Retinopathy Study Chart (logMAR) | Binocular habitual VA: 0.28 ± 0.20  Operated eye habitual VA: 0.48 ± 0.29 | Binocular habitual VA:  0.13 ± 0.20  Operated eye habitual VA: 0.16 ± 0.20 |
| **Feng, 2018** | N = 55 | Early Treatment Diabetic Retinopathy Study Chart (logMAR) | Better eye: 0.18 ± 0.15  Worse eye: 0.39 ± 0.24  Binocular: 0.15 ± 0.15 | After 1^st^ surgery  Better eye: 0.10 ± 0.22  Worse eye: 0.36 ± 0.26  Binocular: 0.08 ± 0.21  After 2^nd^ surgery  Better eye: -0.00 ± 0.19  Worse eye: 0.11 ± 0.19  Binocular: -0.02 ± 0.19 |

* SD: not reported. ** Median (inter-quartile range)
